# Tryptophan Biochemistry: Structural, Nutritional, Metabolic, and Medical Aspects in Humans

**DOI:** 10.1155/2016/8952520

**Published:** 2016-01-12

**Authors:** Lionella Palego, Laura Betti, Alessandra Rossi, Gino Giannaccini

**Affiliations:** ^1^Department of Clinical and Experimental Medicine, University of Pisa, 56126 Pisa, Italy; ^2^Department of Pharmacy, University of Pisa, 56126 Pisa, Italy; ^3^Interdepartmental Center of “Nutraceutical Research and Food for Health”, University of Pisa, 56124 Pisa, Italy

## Abstract

L-Tryptophan is the unique protein amino acid (AA) bearing an indole ring: its biotransformation in living organisms contributes either to keeping this chemical group in cells and tissues or to breaking it, by generating in both cases a variety of bioactive molecules. Investigations on the biology of Trp highlight the pleiotropic effects of its small derivatives on homeostasis processes. In addition to protein turn-over, in humans the pathways of Trp indole derivatives cover the synthesis of the neurotransmitter/hormone serotonin (5-HT), the pineal gland melatonin (MLT), and the trace amine tryptamine. The breakdown of the Trp indole ring defines instead the “kynurenine shunt” which produces cell-response adapters as L-kynurenine, kynurenic and quinolinic acids, or the coenzyme nicotinamide adenine dinucleotide (NAD^+^). This review aims therefore at tracing a “map” of the main molecular effectors in human tryptophan (Trp) research, starting from the chemistry of this AA, dealing then with its biosphere distribution and nutritional value for humans, also focusing on some proteins responsible for its tissue-dependent uptake and biotransformation. We will thus underscore the role of Trp biochemistry in the pathogenesis of human complex diseases/syndromes primarily involving the gut, neuroimmunoendocrine/stress responses, and the CNS, supporting the use of -Omics approaches in this field.

## 1. Introduction

L-Tryptophan (L-Trp) is a large neutral amino acid (LNAA) present in living organisms, precisely one of the 20 L-amino acids (AAs) incorporated in proteins during the process of mRNA translation. All Trp residues in protein and peptide sequences are conventionally indicated with the alphabetic letter W. The AA L-Trp, discovered by the English chemist F. Hopkins in 1901, is also one of the 9 essential AAs for humans which cannot be endogenously synthesized and need to be supplied with aliments, as revealed through diet manipulation studies [1]. Besides being an intermediate of protein/peptide synthesis and turn-over, Trp is the object of scientific investigations in human biological research since decades because of its transformation, after absorption, into a series of small bioactive, pleiotropic compounds, each capable of influencing a number of cell metabolic pathways and physiological responses. Hence, alterations of L-Trp-deriving compounds can be found associated with a variety of metabolic diseases and syndromes affecting those systems and organs responsible for maintaining the chemical, cellular, and behavioural homeostasis: the gut-liver apparatus and the neuroendocrine and immune systems along with the CNS. In particular, an imbalanced metabolism of this AA can interfere with the ability of these systems to interact with as well as discriminate, during development, stressors and stimuli, exogenous and endogenous antigens, and nutrients and xenobiotics.

Amongst Trp-derived compounds produced in the human body, there is the ancient neurotransmitter serotonin (5-hydroxy-tryptamine, 5-HT), a biogenic amine which is known to regulate, in the human CNS, the main adaptive reactions and responses to environmental changes, such as mood-anxiety, cognition, nociception, impulsivity, aggressiveness, libido, feeding behaviour, and body temperature [2, 3]. Next to its role as a neurotransmitter, 5-HT also modulates the activity of peripheral districts, in particular the gut function, the immune and inflammatory responses, the differentiation process of blood stem cells, and the hemodynamic function [3]. Indeed, an altered 5-HT transmission has been found associated with mood-affective disorders [4], autism and cognitive deficit [5, 6], anorexia or bulimia nervosa and obesity [6], and other syndromes presenting peripheral symptoms, such as fibromyalgia, chronic fatigue syndrome, and irritable bowel syndrome (IBS) [7]. Moreover, 5-HT is in turn the precursor of the circadian regulators N-acetyl-5-HT (NAS) and melatonin (MLT), primarily produced in the pineal gland but also in periphery where the two indoleamines act as scavenger compounds [8]. In vertebrates and humans, another main metabolic pathway of Trp is the indole ring breakdown, through the so-called “kynurenine shunt” which produces a number of molecules involved in inflammation, immune response, excitatory neurotransmission, and many other functions.

Only a very small amount of endogenous/dietary L-Trp is converted into 5-HT, suggesting that the bioavailability of this AA and/or changes in the regulation of its metabolism in tissues might be critical for maintaining a healthy balance between all its different paths and destinies [9, 10]. Even though the various components of L-Trp metabolism have been studied since a long time, their regulatory mechanisms in humans have been explored in a lesser extent, especially concerning developmental and/or gender-dependent aspects. Without claiming to provide herein an exhaustive vision of the complexity of Trp research in living organisms and humans, we will start this review by highlighting the impact of the chemistry of this molecule for life, its distribution in the alimentary chain, and nutritional value for human diet and then presenting some among the main tissue-dependent mechanisms of Trp uptake/metabolism. We will then underpin those molecular players in Trp biochemistry which are considered or are possible vulnerability markers in the physiopathology of human complex diseases, trying to point out their regulation. At the same time, we will briefly introduce some Trp research targets actually under investigation for therapeutic strategies in human pathology as well as the utility of -Omics approaches.

## 2. Tryptophan: A Pivotal Chemical Structure for Living Organisms

**Figure 1:**
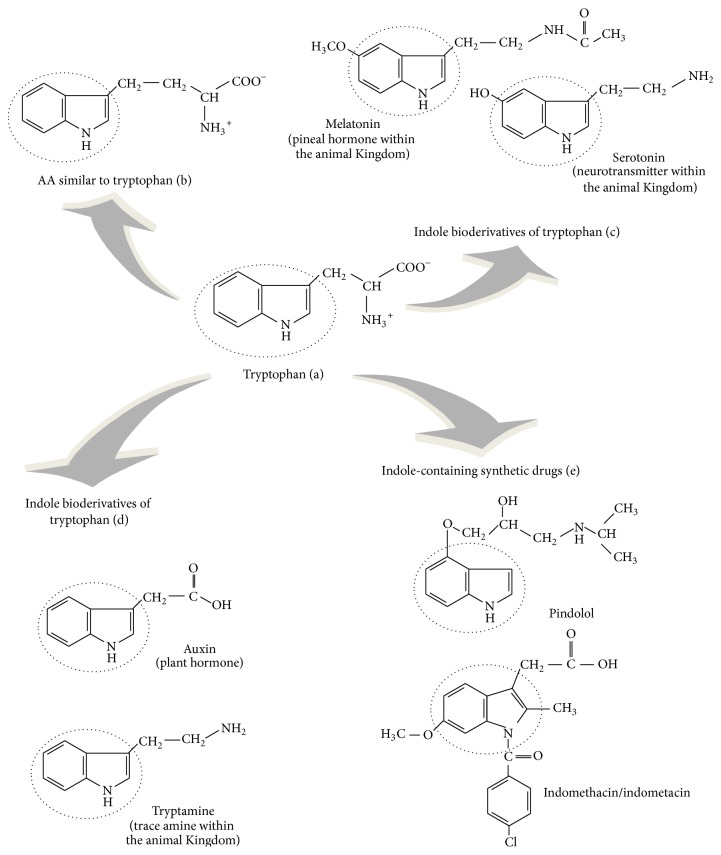
Tryptophan and other indole-containing compounds.

The molecular evolution of life in Earth has selected the chemical structure of –R groups of the 20 L-AAs as the most suitable for building proteins. L-Trp is the only AA in proteins deriving from indole, a bicyclic ring formed by a benzene and a pyrrole group (Figure 1(a)), linked to the *α*-carbon by a –CH_2_-group. The presence of the indole ring in the chemical structure of Trp gives high hydrophobic features to this molecule among all protein AAs. Several AAs could be theoretically synthesized starting from indole (Figure 1(b)), but, amongst these, only L-Trp has been “retained” as a constituent of proteins in living organisms, presumably being the most simple structure of all possible indole AAs. In fact, Trp is the AA at the highest number of C atoms (C_11_) and the presence of other C atoms or substituent groups would be unnecessary. The advantage to keep indole in life chemistry derives either from the possibility to exploit its C_11_ skeleton in metabolism or to utilize it as –R residue in proteins and peptides to promote and stabilize their structure. Also, Trp is metabolized to produce biologically active indole compounds which have great impact on life functions. In fact, beside being present in the chemical structure of the neurotransmitter 5-HT and, in turn, in the circadian molecules NAS and MLT in animals and humans (Figure 1(c)), the indole ring of Trp can be transformed into bioactive compounds also by plants: for instance, the plant hormone indole-3-acetic acid (IAA) or auxin, the defense compounds indolyl glucosinolates [11], and the indole alkaloid and natural hallucinogen dimethyltryptamine. In particular, the plant hormone auxin has been found linked to a specific Trp metabolism pathway involved in plant photoperception and development [11, 12]. Interestingly, indoleamines as 5-HT and MLT have been detected also in plants where their function is under investigation [13]. Similarly, tryptamine (Figure 1(e)) and derivatives, as dimethyltryptamine, have been found in mammalian CNS where they act as neuromodulators, the so-called “trace amines” [14]. Thus, the physiological significance of indole derivatives and their pathways in the evolution of living organisms pivotally involves development, the response to light and environmental variations, and defence against viruses, bacteria, parasites, and/or toxic substances.

Moreover, the study of the metabolic fates of Trp and the other aromatic AAs (AAAs) phenylalanine (Phe) and tyrosine (Tyr) in living organisms has revealed that these pathways represent a “signature” of the cellular evolutionary stages, starting from prokaryote unicellular organisms, passing through eukaryote ancestor cells, arriving then to distinct vegetal or animal eukaryote cells with specific subcellular compartments and trophic features [15]. If Trp is essential for animals, bacteria or other eukaryotes as fungi and plants are instead able to synthesize it from chorismic (also the precursor of Tyr and Phe) and anthranilic (Trp path only) acids. In bacteria, fungi, and plants, the biosyntheses of Trp, Tyr, and Phe are linked together by the shikimate pathway: phosphoenolpyruvate (PEP) and erythrose 4-phosphate (E-4P), deriving from glycolysis and the pentose phosphate shunt, enter into a series of reactions involving the activity of seven enzymes, whose final product is chorismate, the common precursor for the synthesis of the other two main metabolites, prephenate and anthranilate, the first generating Tyr and Phe, the last producing Trp. Prephenate derives from chorismate through the activity of the enzyme chorismate mutase; in turn, prephenate enters into a 3-branch path producing Tyr and Phe. The biosynthesis of Trp in bacteria shares common genes and chemical reactions with plants or fungi: chorismate is recognized by the enzyme anthranilate synthase which transfers to it an amino group from the AA glutamine generating anthranilate and pyruvate; anthranilate is then transformed into Trp via 5 subsequent enzymatic steps. Bacteria and plants or fungi follow, however, different regulatory mechanisms of this metabolic path. In bacteria, chorismate produces Trp under the control of one of the most studied models of gene expression regulation in prokaryote organisms, the Trp operon, activated or repressed depending upon the intracellular concentrations of this AA: operon genes are not constitutively expressed but are induced only by the absence of Trp [16, 17]. The regulation of Trp formation then diverges from the bacterial one because of the different gene organization in eukaryotes. In the absence of the operon, the biosynthetic path of this AA is essentially regulated through a negative feedback of the final product on anthranilate synthase, whose *α*-subunit is specifically recognized by Trp [15]. Additionally, Trp formation in plants has been found tightly regulated by several transcriptional factors acting on gene expression of the enzymes of the shikimate pathway and AAA metabolism, as evidenced in the Brassicaceae* Arabidopsis*. Many of these transcriptional factors have been identified, each differentially stimulated by diverse stressors, as infections by pathogens, trauma, or light. In particular, different stressors can selectively induce the expression of genes involved in Tyr/Phe or Trp biosynthesis, suggesting a distinct physiological significance of the two branches of AAA metabolism in plants [18, 19]. In the common ancestor of animal and vegetable cells, the shikimate path was localized in the cytosol whereas in higher plants this metabolic shunt occurs inside plastids [15]. Thus, genes of Trp biosynthesis would have been lost in plastid-lacking animal eukaryote cells. These considerations lead to reasonably think that AAAs metabolism and production of their bioactive derivatives occupy a central position in the early stages of the evolution of living organisms and trophism lineages. It is not therefore surprising that AAAs represent foremost compounds for human nutrition and health. The importance of maintaining intact the indole ring in Trp derivatives is mirrored by natural therapeutic agents: plant indole alkaloids, as vinblastine and related compounds, exert in fact antitumor properties. Also, indole derivatives have been used in pharmacological research as the starting point for the synthesis of therapeutically relevant compounds. These comprise the nonsteroidal anti-inflammatory compound indomethacin and the antihypertension drug pindolol, a *β*-adrenoceptor blocker acting also on 5-HT transmission (Figure 1(e)).

## 3. Tryptophan Residues in Proteins and Peptides

The presence of Trp residues in polypeptides, as previously introduced, deserves a specific mention: in fact, the Trp indole ring in –R residues gives unique properties to proteins and peptides promoting protein-protein, protein-peptide, or protein-biomolecule structural hydrophobic interactions. The Trp indole ring is able to stabilize structures, domains, and interactions through Van der Waals forces while the indole-N shows propensity as a hydrogen bond donor evidencing a role of this AA also in protein binding and recognition. The presence of Trp –R groups in precise domains, for instance, in transmembrane domains of membrane-bound proteins, is fundamental for the protein stability/assemblage to the phospholipid bilayer [20]. Hydrophobic interactions between proteins and peptides or between these and other biologically active molecules have great importance in cell physiology. Some reviews in the current literature show interesting investigations focusing on these structural aspects of Trp residues: these works are relevant in the study of both cellular and synthetic (peptidomimetics) peptides, with the purpose of evaluating the specific function of secondary and tertiary conformational structures [21]. It is noteworthy that protein hot-spots relevant as therapeutic targets are frequently localized in Trp-rich *β*-hairpin regions [21]. Residues of Trp in the AA sequence of small bioactive peptides, as endogenous anti-inflammatory/antiobesity melanocortin peptides or defense antimicrobial peptides of innate immunity [22, 23], need to be further explored in the field of peptides' structure-activity relationships.

## 4. Tryptophan Requirement and Content in Food

A main consideration deriving from previous paragraphs is that Trp is precious for life: its biosynthetic pathway is in fact energetically expensive and requires the expression of several enzymes and substrates either for Trp operon in bacteria or for the shikimate and chorismate paths in plants. This probably explains why L-Trp is an AA scarcely represented in the alimentary chain [21, 24–26] and its presence in animal cells and tissues must be tightly regulated. The frequency of Trp residues in proteins is, on average, 1-2% with respect to 5% of other AAs and 9% of leucine, the most abundant AA [27]. The recommended dietary Trp daily doses for human adults ranges from 250 to 425 mg/day, corresponding to 3.5–6 mg kg^−1^ (meanly 4 mg kg^−1^) body weight per day [27, 28]. As for other essential AA, new-borns and children require from the diet much higher Trp levels than adults, about 12 mg kg^−1^ bw day [28]. Together with cysteine (Cys), Trp is the essential AA required in lesser amount in human diet [27, 29]. This apparent paradox, due to the variety of important enzymatic reactions in the body and production of crucial metabolites deriving from Trp, would suggest that just a “right” amount of this AA is necessary for humans, without a need to be accumulated: its chemistry is necessary for health but not its accrual in tissues.

The AA Trp is introduced along with all other AAs in the body with protein-rich foods, mainly of vegetal or animal origin. Aliments at higher Trp content include animal origin: milk, cheese, and dairy products, eggs (white), meat, and seafood (fish and crustaceous) and vegetal origin: potatoes, chickpeas, soybeans, cocoa beans, and nuts (walnuts, hazelnuts, and cashew). Lower Trp amounts can be found in some varieties of cereals and maize. Thus, a normal, varied, and balanced diet, as in developed countries, can largely ensure the daily Trp requirement. A main nutritional impact of Trp for human diet is represented by chronic exposure to a diet low in niacin (vitamin B_3_) and Trp, which produces pellagra, a metabolic dysfunction defined by severe alterations of the skin, gut, and brain activity [30]. Pellagra was frequent in past centuries, in people eating almost exclusively low-niacin/Trp maize varieties. In fact, niacin is classified as a vitamin, but this compound can be produced through the metabolic transformations of L-Trp into its precursor quinolinic acid; this explains why L-Trp exerts a protective action against the onset of pellagra symptoms in low-niacin diets. Thus, in economically disadvantaged countries, Trp content in foods, together with other essential AAs, can be of great importance. The analysis of the composition of nutrients, vitamins, essential elements, and AAs represents the basis for good health and children development in these countries. Besides, the amount of Trp in diet represents a challenge for human health and nutritional status worldwide, especially as concerns the regulation of its concentration in plasma as well as its uptake to tissues and brain. The role of the gut microbiome is also an interesting aspect that is emerging as a link between nutrition, gut absorption, Trp fates, and health.

## 5. Tryptophan Absorption, Transport, and Uptake: Regulation of Plasma Levels by Diet, Hormones, and Carriers

On the whole, plasma levels of Trp undergo regulatory mechanisms comparable to those operating for other protein L-AAs: AA uptake occurs in all tissues and cells according to the need for protein synthesis or degradation, with gut, liver, and muscle tissue primarily involved in its modulation. Once introduced with food, all AAs, including Trp, are absorbed by the gut, pass into the bloodstream, are transported to all main tissue districts, overall muscles, and liver, and are finally taken by cells to be part of the AA pool used for the synthesis and turn-over of proteins. Proteolysis and protein catabolism inside cells regenerate, in part, the intracellular reserve of AAs (and Trp) for subsequent protein synthesis and, in part, provoke their release in the bloodstream. Insulin, glucagon, and cortisol are the regulatory hormones of endogenous protein turn-over: insulin blocks the proteolytic activity and promotes recovery of AAs from the bloodstream for protein synthesis in the tissues (overall muscle), while glucagon reduces plasmatic AAs, as alanine (Ala), glycine (Gly), and proline (Pro), for use in the synthesis of glucose in the liver. Cortisol increases the AAs plasma levels (efflux from muscle), shifting the balance towards proteolysis [31]. At the same time, each AA can undergo its own regulation originating its own cell in- and out-flow, in relation to AA composition of both endogenous proteins and those derived from the diet; these last at more variable content [32]. Also, multiple factors as age, gender, or physical activity concur to affect plasma concentrations of AAs [32].

Differently from nonessential AAs, for which, in addition to diet, the rate of* de novo* synthesis is able to control the homeostatic balance of endogenous contents, essential AAs and Trp plasma concentrations are more directly related to their amount in diet. Specifically as concerns dietary Trp, it must be also pointed out that the degree of its relative contribution to protein synthesis/degradation remains unclear [33]: the “paradox” of its rarity in the biosphere and its concomitant worth for life influences its absorption, transport in the bloodstream, tissue uptake and, as a consequence, its destinies.

A foremost and intriguing aspect of human Trp biology is in fact defined by the observation that diet and the type of meal can change its plasma levels as well as its uptake by different cell types. After food digestion, for gut absorption this AAA shares its passage across enterocytes with other neutral AAs through two distinct carrier molecules: the first, expressed at the level of apical membranes of the gut epithelium, is a Na^+^-dependent transmembrane protein codified by the SLC6A19 gene, while the second, named TAT1, is codified by the SLC16A10 gene and is localized on basolateral epithelial membranes, controlling in particular the absorption of AAAs [33]. Tryptophan has also the lowest affinity for the apical carrier than other competitive NAAs, except lysine (Lys), confirming that tissues require defined amounts of Trp and suggesting that gut absorption is a regulated step for the subsequent transport and biotransformation of this AAA.

A widely studied model of Trp uptake mechanism is that regulating its transport across the blood-brain barrier (BBB). For that, insulin and other large neutral AAs, valine (Val), leucine (Leu), isoleucine (Ileu), Tyr, and Phe, have been found to play a chief role: in fact, LNAAs compete with each other for the same transporter system across the BBB, under the control of insulin. This explains why a protein-rich meal increases Trp plasma levels but not its uptake to the brain. Trp uptake to CNS is thus rather favoured by carbohydrate-rich meals. After a carbohydrate meal, 5-HT biosynthesis in raphe nuclei is increased. This mechanism has been extensively studied in mammals: carbohydrate ingestion increases insulin secretion and the clearance of AAs from plasma, in particular of branched-chain AAs (BCAAs: Val, Ileu, and Leu), transported from the bloodstream to muscles, thus increasing Trp availability for CNS uptake and, as described later, to 5-HT synthesis. The 5-HT release at the hypothalamic level activates specific 5-HT receptor subtypes devolved to inhibit appetite brain nuclei [34]. Thus, meal composition, palatable food, and poor protein foods all contribute to Trp uptake across the BBB in favor of 5-HT synthesis. Protein-rich foods in fact contain Trp, but at lower levels than other LNAAs, which, on the whole, rather provoke inhibition of Trp brain uptake. Briefly: a protein-rich meal increases the availability of AAs and Trp, but LNAA competition for transport to the brain reduces Trp entering into the brain in comparison with the amount crossing the BBB after a low protein diet. On the other side, some proteins containing a higher Trp/LNAAs ratio than others, as the milk-derived *α*-lactalbumin, can on the contrary elevate Trp uptake into the brain. Thus, conclusively, proteins can enhance 5-HT synthesis but in relation to their low or high content in Trp [35, 36]. Another important uptake regulatory aspect is represented by the fact that Trp is highly lipophilic and scarcely soluble in aqueous solutions at the physiological pH, so that its transport in blood requires plasma albumin binding: Trp is the only AA transported by albumin. Therefore, a finely regulated equilibrium between free and bound Trp levels exists in plasma, an argument of actual scientific interest and debate.

Next to nutritional considerations, for a deeper understanding of Trp uptake, the transport proteins across tissues and the BBB are currently under investigation. The molecular complex acting as the Trp and other LNAAs transporter across the BBB is formed by a protein which belongs to the superfamily of AA carriers of type “L,” LAT1, and an accessory protein, the cell surface antigen CD98 (heavy chain 4F2), highly expressed in the barrier capillary endothelium [37, 38]. Regulatory mechanisms of this LNAA carrier complex are therefore important for Trp passage into SNC and availability for brain metabolism. The ratio between free and albumin-bound Trp has been also found to modulate Trp passage into the brain: under various conditions, including intense sport activity, activation of nervous sympathetic system, lipolysis, and increased plasma levels of free fatty acids (NEFA), the intracerebral Trp uptake is facilitated by the displacement of the Trp-albumin bound provoking, as a result, the increased availability of the free AA [39]; the Trp-albumin bound is also displaced through interactions with the capillary vessel endothelium (glycocalyx) [40]. Lastly, once it crossed the BBB, Trp can enter inside SNC cells by transport carriers for AAs, which have not been fully characterized. Transport proteins candidates for this role are proteins of the superfamily of G transporters or “ATP-binding cassette transporters,” highly expressed by 5-HTergic neurons. If transporters of types “L” and “G” display an affinity for AAs ranging from 10 and 100 *μ*M, other carriers show a greater affinity for Trp, <1 *μ*M: these proteins are type “T” carriers of pinealocytes or macrophages. This reveals that Trp uptake follows a tissue-dependent regulation based upon a molecular heterogeneity of Trp protein carriers in various tissues [41].

## 6. Tryptophan and Metabotropic G-Protein Coupled Receptors for Aromatic AAs

Some AAs, as *γ*-aminobutyric acid (GABA) or glutamate, are neurotransmitters through the activation of specific subtypes of G-protein coupled receptors (GPCR), particularly relevant for brain function. Research in metabotropic receptors has evidenced a surprising diversity of these proteins and their ligand specificity, also involving elements, nutrients, and metabolites [42]. An interesting aspect needing to be deepened, linked to the topic of this review, is that AAAs and, in particular, L-Trp and L-Phe recognize and activate a class of Ca^2+^ “sensor” and taste metabotropic receptors whose physiological role is under investigation [43]. Beside their primary action on Ca^2+^ regulation, the high expression of these receptors in the gut implies a main action on feeding, food choice, nutrient absorption, and gastrointestinal function [44–47]. It can be supposed that the extensive study of their localization, gene expression, and function within the body would provide useful information and clinical application.

## 7. Metabolic Fates of Trp

**Figure 2:**
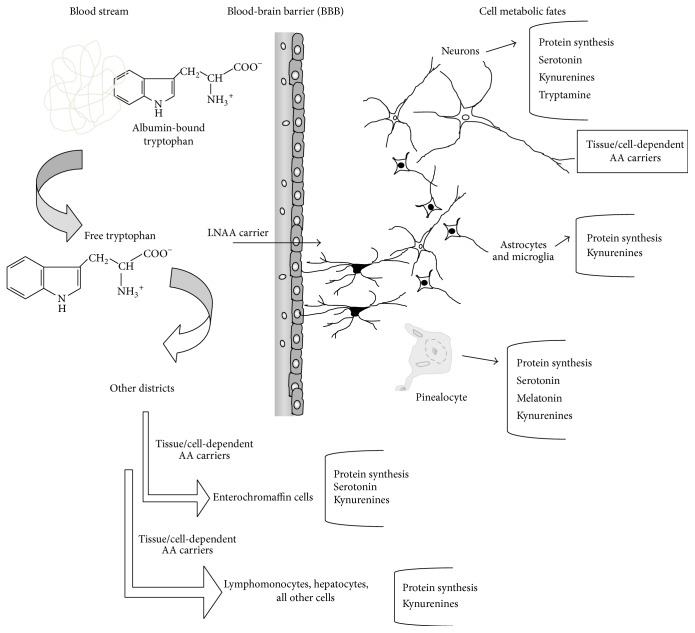
Tryptophan uptake to tissues and main metabolic destinies in either physiological or pathological states.

After its uptake into the various districts, tissues, and cells, Trp is introduced into protein metabolism and synthesis or can enter into various metabolic paths depending upon the tissue expression of specific enzyme activities.  Figure 2 summarizes Trp transport in the bloodstream, its uptake to different tissues, and its main metabolic fates. In substance, beside protein turn-over, Trp metabolism can be divided into two main branches: one, limited to approximately the 3–10% of these Trp biotransformations, which keeps the indole ring intact while producing chemical messengers as the indoleamines 5-HT, NAS, and MLT and the trace amine tryptamine and derivatives and the other, the prevalent one (about 90% or more), which breaks the indole ring generating the kynurenine path, kynurenines, nicotinic acid, and the nicotinamide adenine dinucleotide (NAD^+^) synthesis. We thus will follow herein this schema for describing the Trp metabolic paths which generate low-molecular weight derivatives. The limiting enzymatic reaction for 5-HT biosynthesis is Trp-hydroxylase, TPH, which is active in specialized tissues: 5-HTergic neurons of mesencephalic raphe nuclei; pinealocytes in the pineal gland; blood cells (lymphomonocytes, macrophages, and mast-cells); enterochromaffin cells; neuroendocrine epithelial cells in the lung [41] and other emerging tissues. The synthesis of 5-HT occurs in two enzymatic steps: the first consists in the C-5 hydroxylation of Trp at the level of the benzene ring of the indole (cofactors: O_2_ and tetrahydrobiopterin, TBH_4_) by TPH leading to 5-hydroxy-Trp; the second one is the decarboxylation of 5-hydroxy-Trp to 5-HT, a reaction catalyzed by the enzyme L-amino acid aromatic decarboxylase (cofactor: pyridoxal-5′-phosphate, P5P). This last enzyme is ubiquitous. Newly synthesized 5-HT can enter into storage vesicles to be released as a neurotransmitter in CNS or a modulator in periphery; after its release, excess 5-HT is internalized again through 5-HT reuptake (5-HT transporter, SERT), degraded to 5-hydroxy-acetaldehyde by monooxygenase activities (MAO-A) on mitochondrial outer membrane and then oxidized into 5-hydroxyindoleacetic acid (5-HIAA) by aldehyde dehydrogenase (cofactor: NAD^+^). This last compound is excreted in urine. Two main TPH isoforms exist, TPH_1_ and TPH_2_, codified by distinct genes [48]. The TPH_1_ activity is prevalent in periphery and pinealocytes, while TPH_2_ is expressed in raphe nuclei. Both isoforms are partially saturated in tissues, so that the rate of 5-HT production depends on Trp levels in the SNC and periphery. Intriguingly, in pinealocytes, specialized in the production and secretion of the circadian hormone MLT, the precursor 5-HT can be obtained either by its uptake through a pineal 5-HT transporter (SERT) [49] or by its synthesis from Trp, transported by type “T” protein carriers [50]. Then, 5-HT is acetylated by the enzyme aryl-alkyl-amine-N-acetyl transferase (AANAT) producing NAS which is in turn converted by the enzyme hydroxyl-indole-methyl-transferase (HIOMT) and cosubstrate S-adenosyl-methionine (SAM) into MLT. Melatonin synthesis can also occur in peripheral tissues where this molecule acts as a paracrine/scavenger effector. Another metabolic fate which maintains the indole ring is the formation, by Trp direct decarboxylation, of the trace amine tryptamine, a compound with a physiological meaning which has not been fully understood. Trace amines, present in mammalian tissues at very low, nanomolar, concentrations, can be divided into those deriving from Trp (5-HT-related) and those deriving from phenylalanine and tyrosine (catecholamine-related) and are thought to regulate monoamine transmission [51]. A class of metabotropic, G-protein coupled receptors (GPCR) specific for trace amines recognition (trace amine associated receptors, TAARs) has been discovered [52]. TAARs have been widespread localized in mammalian brain, prevalently in the amygdale region; they are highly expressed in populations of nonmonoaminergic neurons colocalized with monoaminergic neurons [52], implying that trace amines can exert a GPCR-mediated regulation of monoamine neurotransmission. Interestingly, in humans, TAARs genes have been located in chromosome 6, within a DNA region linked to schizophrenia and bipolar disorder [53]; these receptors (TAAR1), which activate adenylate cyclase and cAMP via a G_S_ protein, have been also found to exert a chief role in drug addictions.

The most active metabolic path of Trp is the indole-breaking pathway, the so-called L-kynurenine shunt. Genes codifying for enzymes of the kynurenine shunt have been prevalently found in eukaryote animal cells. Aerobic bacteria also express enzymes of this path [54], implying the ancient origin of this biotransformation. The majority of studies concerning Trp breakdown have been conducted in animals: the kynurenine pathway is active in almost all tissues, being almost rate-limited by the first reaction, the opening, by oxidation, of the indole ring of this AA: the reaction can be catalyzed by two types of heme-containing enzymes differently located in tissues: (a) the Trp-2,3-dioxygenase or Trp pyrrolase (TDO) mainly expressed in the liver but also in brain, prevalently in populations of astrocytes and (b) the indoleamine 2,3-dioxygenase (IDO) expressed in most peripheral tissues, in immune system cells and in the SNC, prevalently in microglia [41, 55, 56]. If TDO is substrate-specific, IDO recognizes and disrupts indole also from D-Trp, 5-HT, MLT, 5-HIAA, and tryptamine. Metabolites deriving from all the other reactions of the kynurenine shunt are considered either cytoprotective (kynurenic acid) or cytotoxic/proepilepsy (3-hydroxy-kynurenine, 3-hydroxy-anthranilic, quinolinic, and nicotinic acids). The kynurenic acid is an antagonist of the excitatory neurotransmitter N-methyl-D-aspartic acid (NMDA) modulating the synthesis of antioxidant species and MLT; the nicotinic path, formed by NMDA agonists, concurs to NAD^+^ synthesis and can also generate free radicals. In effect, an adaptive orchestration of each of these derivatives is physiologically relevant: gene expression of IDO, TDO, or other enzymes of this shunt can be in fact differentially and subtly modified/regulated in various physiological conditions, following a dynamic model. The deregulation or malfunctioning of this shunt, for instance, due to a significant enzyme hypo- or hyperactivation, can underlie a pathological state. Also, manipulations of this path can underscore significant cell responses. Secretion of cytokines and proinflammatory factors as *γ*-interferon (*γ*-INF) and tumor necrosis factor-*α* (TNF-*α*) induces IDO gene expression, whereas antidepressant drugs, tricyclic or selective 5-HT reuptake inhibitors (SSRIs), are able to inhibit TDO, while increasing Trp plasma levels and 5-HT synthesis [41, 57, 58]. The balance between the kynurenine shunt, formation of quinolinic acid or other species potentially producing free radicals, and production of antioxidant metabolites is under the control of the purine system. Purines, as NMDA antagonists, counteract Trp catabolism towards the production of prooxidant species under physiological and pathological conditions [59].

## 8. Regulation of Tryptophan Metabolism and Human Diseases

Both exogenous and endogenous factors finely regulate Trp biotransformation. Diet represents one amongst those main factors which influence Trp availability, distribution, and metabolism in different anatomical districts: Trp diet manipulations have revealed brain, digestive apparatus, and gut as “critical” tissues. Dietary Trp manipulation and acute Trp depletion (TD) have contributed to identify patients' vulnerability to depression or other mood symptoms linked to dysfunctional monoaminergic systems: TD strongly modifies Trp plasma levels, decreasing 5-HT synthesis and mood tonus in subjects with a familiarity or history of mood-affective disorders, similarly to what is observed, even if with opposite effects, for sleep deprivation/restriction [60]. This suggests that depressed patients would be less capable of compensating dietary Trp variations with respect to healthy subjects. Tryptophan depletion has also shown to affect gut motility in IBS [61]. An altered homeostasis of the Trp/LNAAs ratio in plasma has been considered the origin of the so-called “carbohydrate craving” syndrome, linked to some types of human obesity and/or feeding behavior disorders, often in comorbidity with depression and bipolar disorder [62–64]. These findings relate Trp availability and metabolism with severe illnesses as metabolic syndrome and diabetes. Other human diseases linked to Trp are those generated by defective carriers at the level of gut absorption, as Hartnup disease, showing pellagra-like symptoms, or malabsorption conditions [65]: fructose malabsorption is characterized by low Trp plasma levels and mild depressive symptoms, evidencing a link between gut dysfunction, Trp availability, and mood. In addition to (i) the influence of diet or alimentary intolerances and/or (ii) Trp content in protein and control exerted by insulin/glucagon, Trp absorption/metabolism in the different cells and tissues can be specifically regulated by many other factors involving the stress neuroendocrine axis, the immune response, and inflammation. A main aspect needing to be better understood is the variation and adaptation of Trp metabolism during the lifespan: the relative contribution of each destiny (5-HT synthesis, tryptamine, kynurenine shunt, and protein synthesis) is supposed to vary during development and aging, in relation to gender, individual vulnerability, and/or lifestyle. The competition between 5-HT synthesis and the kynurenine shunt in cells expressing both enzymatic pools is in fact under the control of various peptides, neurotransmitters, hormones, and cytokines, also in response to stressors of different nature and to variations of the quality of life, an issue still presenting unsolved questions [66]. The enzymatic pool of TPH, TDO, and IDO can be specifically modulated by all these factors: environmental factors and gene polymorphisms/vulnerability can concurrently modify Trp fates. The enzyme TDO is under the control of the arousal response, by cortisol and prolactin [41, 67], implying that, during prolonged stress, 5-HT requirements could be not compensated. The hyperactivation of the glucocorticoid-insensitive IDO can be provoked by cytokines, chemokines, and inflammation mediators. Immune system and inflammation are the main targets of unbalanced IDO activity [68–70]: the role of this enzyme of Trp metabolism consists in the ability of preventing/restraining excessive tissue damage due to cytotoxic immune actions as well as creating a Trp-poor microenvironment as a host defense mechanism. In addition, the activity of IDO is involved in the regulation of Trp levels during development, for instance, to establish immune tolerance and the discrimination of self/non-self-antigens during pregnancy ensuring the homeostasis of individual tissue identity and defense functions [71, 72]. The IDO fine tuning and the *γ*-interferon mediated switching of its isoforms, IDO1 and IDO2, modulate the balance between immune response suppression or activation [73]. It is worth noting that the regulation of these two IDO activities is disturbed in cancer cells, participating in immune tolerance against tumor antigens [73].

Intriguingly, IDO has also a particular relevance in mood disorders and in neurodegenerative SNC diseases, brain aging, and Alzheimer or Parkinson disease [74, 75]. Oxidative stress and TDO and IDO activities have been found impaired in children with autism [76, 77]. Other factors which influence the availability and metabolism of Trp are aging and gender-related mediators, sustaining the prevalence of many pathological conditions in the elderly or in one of the two sexes [78–82]. Feeding disorders, obesity, and depression are more frequent in women and TD has revealed gender-dependent mood vulnerabilities. The homeostatic interrelation between 5-HT-glucocorticoids can vary in different phases of relational/social life, as adolescence in the two sexes, pregnancy, premenstrual or pre- and postmenopausal periods in women, or aging in man and women separately [83]. The onset of feeding behavior disturbances as bulimia or anorexia nervosa is prevalent in women occurring during adolescence, as sustained in homozygote twin studies [84]. Exposition to chronic stress can modify appetite and macronutrient choice. In feeding disorders, the evaluation of Trp plasma levels versus the other LNAAs can provide useful information on either the nutritional state or patient metabolism, as well as the AA availability of brain uptake [85]. Other new potential molecular targets for these disorders are the metabotropic aromatic AA GPCRs [45, 86] or circulating tryptamine levels and TAARs [87]. The measure of the levels of Trp versus metabolites of the kynurenine shunt could permit “visualizing” the adrenal axis activation, proinflammatory cytokine secretion together with levels of the excitatory AA glutamate. On the other side, the genetic research has confirmed the importance of Trp metabolism as a support for a better clinical response and tolerance to treatments, for instance, in clinical psychiatry and SNC pathology: if polymorphisms of the SERT gene and 5-HT or other monoamine receptor subtypes are directly involved in these disorders and their treatment, the presence of polymorphisms of the brain Trp uptake G transporter as well as those related to TPH_2_ has been observed in mood disorders [88, 89], often in comorbidity with bulimia or anorexia. Genetic polymorphisms of the SLC6A14 carrier transport for basic and neutral AAs as Trp have been related with human obesity [90]. Moreover, Trp metabolism components have been linked to gender-dependent disturbances associated with oxidative stress and cognitive processes [91, 92]. In summary, disturbances of Trp metabolism occupy a key position in human pathology and complex, multifactorial diseases.

## 9. Therapeutic Strategies and Drug Development

Therapeutic strategies based on Trp chemical properties are in progress. As already reported, synthetic indole derivatives have been used for treating various human diseases (Figure 1(e)). New compounds are currently studied for human health. For instance, Trp-containing peptides or Trp-containing agents are explored as therapeutic agents against protein aggregation in neurodegeneration processes [21, 93, 94]. Alternative strategies in treating CNS diseases consist instead in targeting the kynurenine shunt and its modulation, acting therefore in the core of the balance of Trp fates: since kynurenine derivatives are related to both NMDA agonism or antagonism and nicotinic acid paths, their metabolism can be evaluated for treating cognitive deficits, dementia, and other severe neuropsychiatric conditions [95].

As reported before, Trp metabolism plays a relevant role in cancer: as regards IDO-related immune tolerance for cancer antigens, methyl-Trp derivatives and IDO inhibitors are promising compounds for therapy against tumor growth and metastasis formation [96]. New hydroxyl-indole derivatives are also appraised for their lactate dehydrogenase (LDH) inhibitory effect and anticancer properties [97].

## 10. Beyond Tryptophan Research: The Usefulness of the -Omics Techniques

L-Tryptophan biochemistry lies in the heart of converging nutritional, neuroendocrine, and immune paths, through a variety of molecular effectors, each presumably contributing to relevant, complex, and severe diseases and syndromes, as reported in previous paragraphs. Advances in technologies of applied biochemistry and molecular biology have much improved the study of Trp metabolism and its implications in clinical research and medical genetics. Actually, new perspectives are emerging: in particular, it appears increasingly evident that pathologies at unclear aetiology/pathogenesis need multidisciplinary and multifactorial proceedings. This would allow defining groups of patients within the same disease showing common and distinct symptoms or responses to treatment correlated with specific biochemical patterns. For instance, the identification of biochemical clusters within neuropsychiatric disorders or other complex diseases would further support the notion that these illnesses are not “single,” “fixed” pathological entities but rather spectrum disorders [98]. The targeting of Trp biochemistry in the context of other metabolic pathways is included in such a methodological advance. This approach would enable explaining symptoms' overlaps such as chronic fatigue and depression, pain disturbances, and IBS [7], sustaining the confluence of vulnerability factors for a complex disease and/or for diverse treatment responses. This research field would also provide useful information for cancer research and therapy. A valuable and consistent help seems to come from high-dimensional biology [99], involving the -Omics tools, as, primarily, the genomics, transcriptomics, proteomics, and metabolomics ones [100]. Metabolomics techniques permit in particular appraising multiple pathways and a number of metabolic intermediates, known and unknown, following an opposite approach of the classical one. This would allow the tracing of metabolic signatures of patients, to evaluate epigenetic and genetic factors which define a pathological condition as well as identify new correlates of pharmacological responses or new susceptibility traits of disease. Another main advantage of these approaches is that they can implement the use of personalized pharmacological therapies, also with respect to patients' age, gender, and lifestyle, by possibly considering diet habits and supplementation with specific nutrients and elements.

## 11. Conclusions

The essential AA tryptophan displays a peculiar chemistry among all other protein AAs and its derivatives are conserved in all living organisms, being linked to stress/environmental adaptive response. In humans, the molecular effectors of its indole-conserving or indole-disrupting fates are up- and downregulated by multiple factors which can play a role in many human complex diseases and syndromes. Molecular biology techniques and genetics are investigating components of Trp pathways while the application of high-dimensional biology and -Omics techniques is supposed to provide more insights about the regulation of Trp content in cells, its availability for human nutrition, and its role in the pathogenesis of disease. Other perspectives in Trp research concern the efficacy, monitoring, and personalization of pharmacological treatments as well as the development of new therapeutic compounds.
